# Trajectories of body mass index before the diagnosis of cardiovascular disease: a latent class trajectory analysis

**DOI:** 10.1007/s10654-016-0131-0

**Published:** 2016-03-08

**Authors:** Klodian Dhana, Joost van Rosmalen, Dorte Vistisen, M. Arfan Ikram, Albert Hofman, Oscar H. Franco, Maryam Kavousi

**Affiliations:** Department of Epidemiology, Erasmus Medical Center, PO Box 2040, 3000 CA Rotterdam, The Netherlands; Department of Biostatistics, Erasmus Medical Center, Rotterdam, The Netherlands; Steno Diabetes Center, Gentofte, Denmark; Department of Neurology, Erasmus Medical Center, Rotterdam, The Netherlands; Department of Radiology, Erasmus Medical Center, Rotterdam, The Netherlands; Department of Epidemiology, Harvard T.H. Chan School of Public Health, Boston, MA USA

**Keywords:** Obesity/physiopathology, Cardiovascular disease, Body mass index, Blood pressure, Lipids, Glucose

## Abstract

Patients with cardiovascular disease (CVD) are a heterogeneous group regarding their body mass index (BMI) levels at the time of diagnosis. To address the heterogeneity of CVD, we examined the trajectories of change in body mass index (BMI) and in other cardio-metabolic risk factors before CVD diagnosis. The study included 6126 participants from the prospective population-based Rotterdam Study, followed over 22 years with clinical examinations every 4 years. Latent class trajectory analysis and mixed-effect models were used to develop trajectories of BMI and other cardio-metabolic risk factors respectively. During follow-up, 1748 participants developed CVD, among whom we identified 3 distinct BMI trajectories. The majority of participants (n = 1534, 87.8 %) had steady BMI levels during follow-up, comprising the “stable weight” group. This group showed decrease in mean high-density lipoprotein (HDL) cholesterol over time. The second group, the “progressive weight gain” group (n = 112, 6.4 %), showed a progressive increase in BMI levels. In this group, mean waist circumference increased, mean HDL cholesterol decreased and mean fasting glucose levels were fluctuating over follow-up. In the third group, the “progressive weight loss” group (n = 102, 5.8 %), BMI levels decreased during follow-up. This group showed a decrease in mean waist circumference and in fasting glucose. In conclusion, the majority of individuals who developed CVD had a stable weight during follow-up, suggesting that BMI alone is not a good indicator for identifying middle-aged and elderly individuals at high risk of CVD. Waist circumference, HDL cholesterol, and glucose trajectories differed between the identified BMI subgroups, further highlighting that CVD is a heterogeneous disease with different pathophysiological pathways.

**Electronic supplementary material:**

The online version of this article (doi:10.1007/s10654-016-0131-0) contains supplementary material, which is available to authorized users.

## Introduction

The association between obesity and cardiovascular disease (CVD) has been well established in observational studies [1, 2]. The causality of this relationship has also been recently reported using a Mendelian randomization approach [3]. Additionally, duration of obesity has been shown to be a risk factor for CVD [4, 5], diabetes [6], and mortality [7], independent of the baseline levels of body mass index (BMI). However, CVD is not only limited to obese individuals, and normal weight or overweight individuals may also experience a cardiovascular event [8, 9]. Consequently, patients with CVD are a heterogeneous group with regard to their BMI levels at the time of diagnosis of CVD. Understanding the heterogeneity of CVD by exploring the distinct patterns of change in BMI levels prior to the diagnosis of CVD might carry important implications for improving disease prevention or treatment. For instance, each trajectory of BMI change prior to CVD could be accompanied by different trajectories of change in other cardio-metabolic risk factors. As such, identification of different population subgroups with similar risk factor patterns might serve to facilitate targeted cardiovascular prevention programs.

One way of exploring this heterogeneity is to group individuals with similar patterns of change in BMI over time through data-driven statistical methods such as latent class trajectory analysis [10]. Latent class trajectory analysis is an innovative statistical method used to identify subgroups (classes) of participants who are homogeneous with respect to the trajectory of one specific risk factor but heterogeneous as compared with other subgroups. Latent class trajectory analysis has recently been applied to study BMI development prior to diagnosis of diabetes [11].

In the current study among a middle-aged and elderly population, we aimed to identify different trajectories of BMI development prior to a cardiovascular event. We also sought to explore the trajectories of concurrent cardio-metabolic risk factors, including blood pressure, lipids, and glucose, within each identified BMI subgroup.

## Methods

### Study population

The Rotterdam Study (RS) is a prospective population-based cohort study. In 1989–1993, the original cohort (RS-I) recruited 7983 (78 % response rate) men and women aged 55 years and older from a well‐defined suburb in the city of Rotterdam, the Netherlands. The participants of the Rotterdam Study have been followed-up for more than 22 years and the clinical data have been collected across five subsequent phases, approximately 4 years apart. Each phase of the study included a home interview followed by two visits at the research center for clinical examinations. Details regarding the objectives and design of the Rotterdam Study have been reported previously [12].

**Fig. 1:**
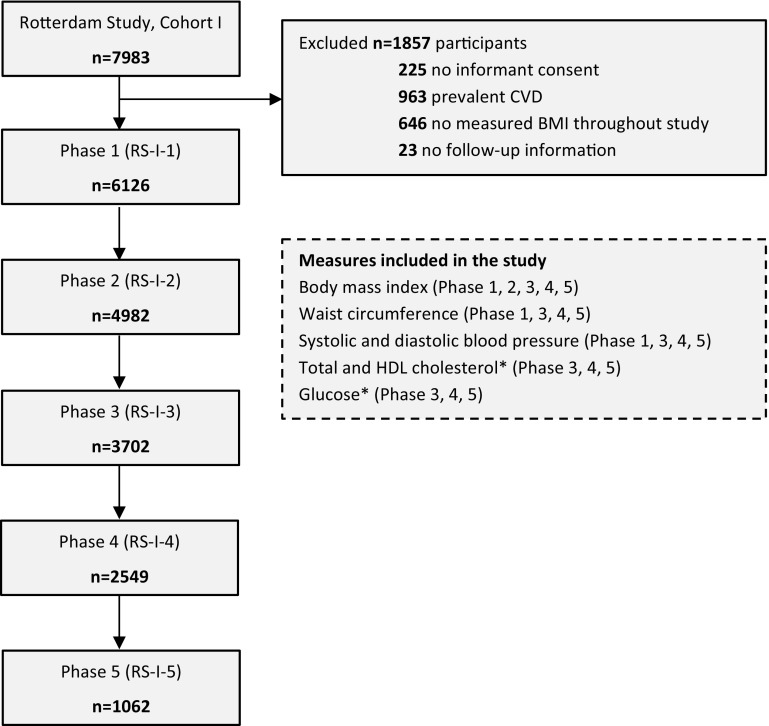
Flow diagram of the participants included at each phase. *HDL* high density lipoprotein, *CVD* cardiovascular disease, *n* number. * Total cholesterol, HDL cholesterol and glucose are measured as fasting in phases 3, 4 and 5

The present analysis was based on the original cohort (RS-I). From 7983 subjects participating at baseline, we excluded 225 participants without informed consent, 963 individuals with prevalent CVD (including: myocardial infraction (MI), coronary heart disease (CHD), heart failure, and stroke), 646 individuals without BMI measurements throughout phases 1–5, and 23 participants without information regarding CVD follow-up. Thus, the final sample included 6126 participants (77 % of original sample) (Fig. 1).

### Assessment of cardio-metabolic risk factors

Information on cardio-metabolic risk factors was collected through home interviews or measured at the study center visit as described previously [13, 14]. Height and weight were measured in all five phases, whereas systolic blood pressure and waist circumference were measured in phases 1, 3, 4 and 5, and fasting total cholesterol, high-density lipoprotein (HDL) cholesterol and fasting plasma glucose were measured in phases 3, 4 and 5 (Fig. 1). Height and weight were measured with the participants standing without shoes and heavy outer garments. BMI was calculated as weight divided by height squared (kg/m^2^). Waist circumference was measured at the level midway between the lower rib margin and the iliac crest with participants in standing position without heavy outer garments and with emptied pockets, breathing out gently. Serum total cholesterol, HDL cholesterol, and glucose were measured using standard laboratory techniques. Blood pressure was measured at the right brachial artery with a random-zero sphygmomanometer with the participant in sitting position, and the mean of two consecutive measurements was used. Smoking status was classified as current smoking or others (former and never) in all phases. We assessed medication use for hypertension, hyperlipidemia and diabetes mellitus through interview.

### Clinical outcome

The main outcome measure was incident CVD [15], composed of coronary heart disease (CHD) [13] and stroke [16]. CHD was composed of fatal and non-fatal myocardial infarction and other CHD mortality. Stroke was composed of fatal and non-fatal stroke. Data on incident CVD were collected through an automated follow-up system and by gathering information from general practitioners working in the study area until January 1, 2012.

### Statistical analysis

Our statistical analysis included two consequent steps. Initially, we used latent class trajectory analysis to identify groups of participants with distinct trajectories of BMI change during follow-up, until the occurrence of the first cardiovascular event [10]. Subsequently, within each identified BMI group, we developed the trajectories of change in other cardio-metabolic risk factors during the follow-up [11].

The latent class trajectory analysis automatically divides the study population into classes, in such a way that participants in the same class tend to have similar trajectories of BMI change. By design of the study we performed this analysis only in the population diagnosed with CVD during follow-up. Therefore, the observation period for the development of trajectories started retrospectively at the date of diagnosis with CVD. For the subjects within each group, the latent class trajectory model assumes that the BMI measurements follow a linear mixed-effects model with BMI as the dependent variable and time before CVD diagnosis (time 0), age, sex and phase of study as independent variables. The independent variable “time before CVD diagnosis” was used to describe the shape of the longitudinal trajectory of BMI, using a cubic specification (i.e., linear, quadratic, and cubic terms for the time before CVD diagnosis were entered as covariates into the model). The Bayesian information criterion (BIC) was used to choose the number of classes in the latent class trajectory model. The latent class trajectory model calculates a posterior probability of membership in each latent class for each participant. Each participant is assigned to the class for which his/her posterior probability is the highest. To ensure that all obtained classes were of clinically meaningful size, we imposed the condition that each class should include at least 5 % of participants and the mean posterior probability of each class should be higher than 75 %. Since the trajectories of change in BMI could differ between individuals who die during follow-up and among individuals who do not die or develop CVD during follow-up [17] we divided the rest of the population into two subgroups: (1) CVD-free and alive until end of follow-up and (2) non-CVD mortality.

For each identified BMI group (among individuals diagnosed with CVD) and the two other groups (CVD-free, and non-CVD mortality), we examined the trajectories in other cardio-metabolic risk factors including waist circumference; systolic and diastolic blood pressure; fasting total and HDL cholesterol; and fasting plasma glucose. Since the aggregated effect of combined risk factors on CVD might differ from each risk factor alone or from merely sum of risk factors, guidelines recommend to evaluate the 10-year CVD risk of an individual in clinical practice [18]. Therefore, we examined the trajectories of 10-year CVD risk in each group of BMI. The predicted 10-year CVD risk was calculated using the American College of Cardiology/American Heart Association (ACC/AHA) Pooled Cohort Equation coefficients, which includes age, sex, total cholesterol, HDL cholesterol, systolic blood pressure, blood pressure lowering medication use, diabetes status, and smoking status in the prediction model [15]. These trajectories of cardio-metabolic risk factors were estimated using linear mixed-effects models. The independent variables in these linear mixed-effects models were follow-up time, age, sex, and study phase. Analyses of lipids were further adjusted for lipid-lowering treatment, analyses of blood pressure were further adjusted for anti-hypertensive treatment, and analyses of glucose were additionally adjusted for diabetes treatment. Quadratic and cubic terms for follow-up time were included in the BMI groups (latent classes) when significant (*p* < 0.05). For individuals not developing CVD during follow-up (CVD-free and non-CVD mortality groups), year 0 is merely a time point in a normal life course, and we therefore fitted the trajectories by using linear models. Pair-wise differences in growth curves between BMI groups were tested using F-tests for each cardio-metabolic risk factor. Paired Chi square test (for categorical variables) was used to compare participant characteristics between the groups. To account for multiple testing due to comparing three pairs of BMI groups, we used a Bonferroni-adjusted significance level of 0.05/3 = 0.0167 for the F-tests for each cardio-metabolic risk factor. All other statistical tests used a significance level of 0.05, and all statistical tests were two-sided.

Analyses were conducted using R statistical software, version 3.0.1 (R Foundation for Statistical Computing, Vienna, Austria), with the package “lcmm” [10].

## Results

Baseline characteristics of the study population are presented in Table 1. Overall, 6126 participants with a mean age of 68.8 years, mostly women (n = 3787, 61.8 %), and overweight (mean BMI = 26.3) were included in the study (Table 1). The median (interquartile range—IQR) of total follow-up was 14.7 (7.6; 18.3) years during which 28.5 % of participants (n = 1748) developed CVD. Among individuals who did not develop CVD, 2184 subjects (35.7 %) remained alive until the end of follow-up and 2194 (35.8 %) died from non-CVD causes. The baseline characteristics of these subgroups are presented in Table S1 in the Supplementary Material.

**Table 1:** Characteristics of study participants at their first clinical examination

Characteristics	Total population (N = 6126)
Time before diagnosis/last visit (years)	14.7 (7.6, 18.3)
Women (%)	3787 (61.8)
Current smoker (%)	1439 (23.5)
Antihypertensive treatment (%)	1035 (16.9)
Anti-diabetic treatment^†^ (%)	240 (6.8)
Statin treatment^†^ (%)	575 (16.3)
Age (years)	68.8 ± 8.9
Glucose^†^ (mg/dl)	105.7 ± 24.2
Cholesterol^†^ (mg/dl)	226.6 ± 37.2
HDL cholesterol^†^ (mg/dl)	54.9 ± 15.6
Systolic blood pressure (mmHg)	139.3 ± 22.1
Diastolic blood pressure (mmHg)	73.9 ± 11.4
Body mass index (kg/m^2^)	26.3 ± 3.7
Waist circumference (cm)	90.0 ± 11.1

*HDL* high density lipoprotein, *CVD* cardiovascular disease, *n* number

* Values are mean ± SD, numbers (percentages), or median (IQR)

^†^Fasting measurements of Lipids and glucose and treatment were available in the third, fourth and fifth visits of the original Rotterdam Study cohort (N = 3529)

### Patterns of BMI change over time

**Fig. 2:**
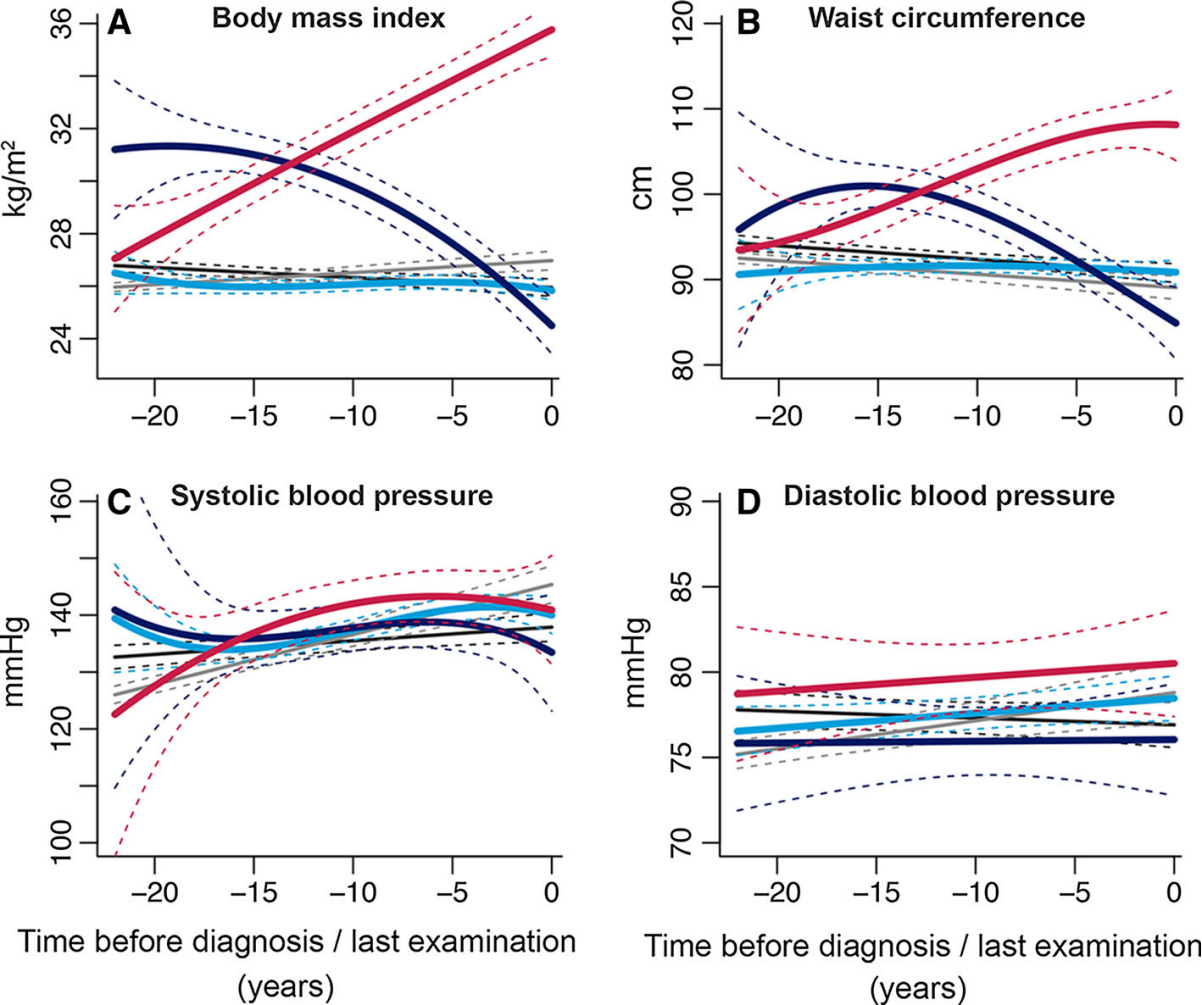
Trajectories of body mass index, waist circumference, systolic and diastolic blood pressure. Trajectories for risk factors during 22 years of follow-up until diagnosis of CVD, death or censoring from the study. The figures represent a hypothetical man of 65 years old. Trajectories for blood pressure represent a person on anti-hypertensive treatment. *Light blue* “stable weight” (including 87.8 % of CVD patients); *dark blue* “progressive weight loss” (including 5.8 % of CVD patients); *red* “progressive weight gain” (including 6.4 % of CVD patients); *gray* “CVD-free”; *black* “non-CVD mortality” groups. Similar trajectories for a hypothetical woman of 65 years of age are shown in Figure S1 in Supplementary Material. (Color figure online)

Among 1748 participants who developed CVD, we identified three groups with distinct trajectories of change in BMI levels. The largest group of individuals (n = 1534, 87.8 %) was classified in the first group and showed stable BMI levels over time. This group entered the study with an average BMI of 25.6 kg/m^2^ and maintained this average BMI level during follow-up. Therefore, we named this group “stable weight”. The second group comprised 112 (6.4 %) individuals who entered the study with a mean BMI of 26.1 kg/m^2^. This group experienced an increase in BMI during follow-up and the mean BMI reached the obesity range (i.e. BMI ≥ 30). We named this group the “progressive weight gain” group. The third group included 102 individuals (5.8 %) who initially started with an average BMI in the obese range (mean BMI = 30.2 kg/m^2^). In this group, the average BMI decreased to the overweight range and continued to decrease for the next 10 years. The mean BMI eventually reached the normal-weight range. We named this group the “progressive weight loss” group (Fig. 2a).

Among 2184 subjects who did not develop CVD event and were alive until the end of follow-up, the “CVD-free” group, the average BMI remained stable. In this group the average BMI ranged from 25.0 to 25.9 kg/m^2^ during the follow-up. Among 2194 subjects who died of other causes during follow-up, the “non-CVD mortality” group, the average BMI at the start of the follow-up (average BMI = 25.8 kg/m^2^) was in the overweight range. This group slightly lost weight during follow-up and just before death their mean BMI was in the normal range (Fig. 2a).

**Fig. 3:**
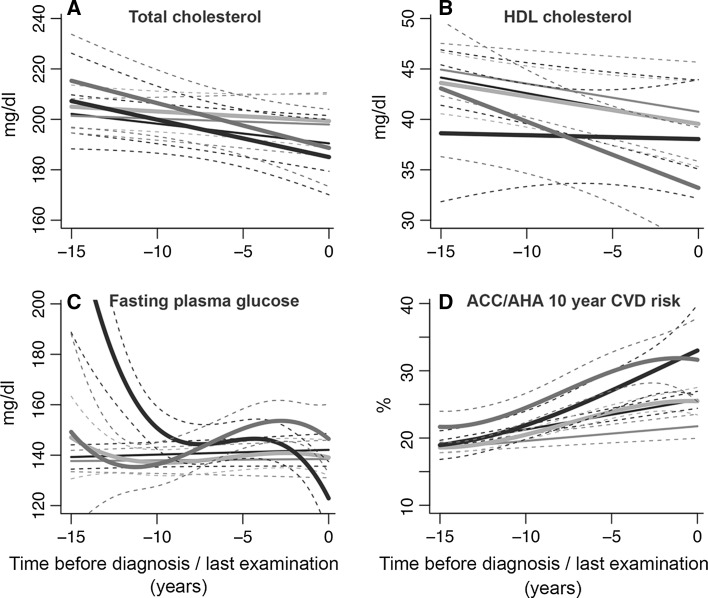
Trajectories of fasting plasma glucose, total cholesterol, HDL cholesterol, and predicted 10-year CVD risk. HDL cholesterol, high-density lipoprotein cholesterol; ACC/AHA, American College of Cardiology/American Heart Association; CVD: cardiovascular disease. Trajectories for risk factors during 15 years of follow-up until diagnosis of CVD, death or censoring from the study. The figures represent a hypothetical man of 65 years, on lipid- or glucose-lowering treatment. *Light blue* “stable weight” (including 87.8 % of CVD patients); *dark blue* “progressive weight loss” (including 5.8 % of CVD patients); *red* “progressive weight gain” (including 6.4 % of CVD patients); *gray* “CVD-free”; *black* “non-CVD mortality” groups. Similar trajectories for a hypothetical woman of 65 years of age are shown in Figure S2 in Supplementary Material. (Color figure online)

While the analyses were performed in the total population, to plot the trajectories of change in BMI and in other cardio-metabolic risk factors, it was necessary to assume a hypothetical individual with a predefined sex and age. Therefore, the presented figures are sex specific. Figures 2 and 3 represent the trajectories for a hypothetical man of 65 years old. Similar trajectories for a hypothetical woman of 65 years of age are shown in Figures S1 and S2 in the Supplementary Material.

### Trajectories of waist circumference

Trajectories of waist circumferences differed significantly between the three groups (*p* < 0.001 for all pairwise comparisons) (Fig. 2b). The trajectories for the “progressive weight loss” and “progressive weight gain” groups broadly resembled the trajectories of BMI in these groups. However, among individuals in the “stable weight” group, we observed a slight increase in the mean waist circumference during follow-up. The mean waist circumference in the “CVD-free” and “non-CVD mortality” groups decreased during follow-up (Fig. 2b).

### Trajectories of blood pressure

Trajectories of systolic blood pressure among “stable weight”, “progressive weight loss”, and “progressive weight gain” groups were not significantly different (*p* ≥ 0.208 for all pairwise comparisons). The “stable weight” and “progressive weight loss” groups had a mean systolic blood pressure between 130–142 mmHg during follow-up. In the “progressive weight gain” group, the average systolic blood pressure levels increased during the follow-up from 120 mmHg to 138 mmHg before the cardiovascular event (Fig. 2c).

Trajectories of diastolic blood pressure for all groups of BMI were in the normal range during follow-up (<80 mmHg). The average diastolic blood pressure trajectory of the “progressive weight gain” group was significantly higher (*p* = 0.012) than the “progressive weight loss” group. Similarly to the “progressive weight gain” group, the “stable weight” and the “CVD-free” groups experienced a modest increase in mean diastolic blood pressure during follow-up. Compared to the “stable weight” and “progressive weight gain” groups, the “progressive weight loss” group had the lowest mean diastolic blood pressure during follow-up. Among the “non-CVD mortality” group, the mean diastolic blood pressure decreased during follow-up and before death (Fig. 2d).

### Trajectories of lipids and glucose

We found no differences in fasting total cholesterol levels between the three groups of individuals who developed CVD during follow-up (*p* ≥ 0.059 for all pairwise comparisons). Overall, the trajectories of fasting total cholesterol followed those of BMI for the “stable weight” and “progressive weight loss” groups. However, the “progressive weight gain” group showed a decrease in average levels of total cholesterol. In this group, the mean total cholesterol levels decreased from 218 mg/dl to 190 mg/dl during follow-up. The average levels of total cholesterol (mean: 200 mg/dl) were stable for the “CVD-free” group (Fig. 3a). Average levels of HDL cholesterol for the three groups of “stable weight”, “progressive weight loss” and “progressive weight gain” were lower compared to the “CVD-free” group. The mean levels of HDL cholesterol decreased significantly in the “progressive weight gain” and the “stable weight” groups during follow-up. The decrease in mean HDL levels was more pronounced in the “progressive weight gain” group compared to the “stable weight” group (*p* < 0.001) (Fig. 3b).

Trajectories of fasting glucose differed between the three groups of “stable weight”, “progressive weight loss” and “progressive weight gain” (*p* < 0.001 for all pairwise comparisons). The “stable weight” group had an average fasting glucose level of 140 mg/dl, which remained stable during follow-up. Among the “progressive weight gain” group, the mean fasting glucose levels were fluctuating (increasing and decreasing) over the entire follow-up. For the “progressive weight loss” group we observed a decline in mean levels of fasting glucose from 200 mg/dl to 120 mg/dl during follow-up (Fig. 3c).

### Trajectories of estimated 10-year CVD risk

Figure 3d shows the 10-year predicted risk of CVD estimated by the ACC/AHA Pooled Cohort Equations algorithm [15]. Despite the differences in BMI trajectories, the 10-year predicted risk of CVD increased similarly during follow-up for all 3 groups of CVD patients. However, the “progressive weight gain” group had significantly higher mean 10-year predicted risk of CVD than the “stable weight” group (*p* < 0.001). In the “progressive weight loss” group, the mean predicted CVD risk increased rapidly 10 years before the CVD diagnosis. The 10-year predicted risk of CVD for the “CVD-free” group was lower than the 10-year predicted CVD risk in all other groups.

### Other characteristics

Table 2 shows characteristics of the participants at the time of CVD diagnosis for the “stable weight”, “progressive weight gain” and “progressive weight loss” groups; or at the last examination for the “CVD-free” and “non-CVD mortality” groups. Compared to the “CVD-free” group, other groups were older and included more men. Individuals in the “stable weight” group were less likely to receive treatment for hypertension and lipids but more likely to smoke compared to the other two groups of “progressive weight loss” and “progressive weight gain”. Interestingly, the proportion of participants with a family history of MI or stroke was significantly lower in the “stable weight” group compared to the “progressive weight gain” group.

**Table 2:** Characteristics of study participants at the time of the diagnosis for the three groups with cardiovascular disease or at last visit for the groups without cardiovascular disease (N = 6126)

Characteristics*	Individuals developing cardiovascular disease during follow-up (n = 1748)			Individuals free of CVD during follow-up (n = 4378)	
	Stable weight (n = 1534)	Progressive weight gain (n = 112)	Progressive weight loss (n = 102)	CVD-free (n = 2184)	Non-CVD mortality (n = 2194)
Age at diagnosis/last contact (years)	75.7 ± 8.1	75.6 ± 7.3	79.4 ± 8.3	75.1 ± 7.1	77.2 ± 8.0
Women (%)	868 (56.6)	75 (67.0)^†^	66 (64.7)	1496 (68.5)^†^	1282 (58.4)
Ever smoker (%)	925 (60.3)	59 (52.7)	49 (48.0)^†^	885 (40.5)^†§^	1202 (54.8)^†^
Ever on antihypertensive treatment (%)	391 (25.5)	58 (51.8)^†‡^	47 (46.1)^†^	662 (30.3)^‡§^	570 (26.0)^§^
Ever on anti-diabetic treatment (%)	141 (9.2)	18 (16.1)	15 (14.7)	170 (7.8)^‡§^	143 (6.5)^†‡§^
Ever on statins treatment (%)	133 (8.7)	26 (23.2)^†^	14 (13.7)	460 (21.1)^†^	145 (6.6)^‡§^
Family history for myocardial infarction or stroke (%)	845 (55.1)	76 (67.9)^†^	62 (60.8)	1232 (56.4)^§^	1143 (52.1)^§^

*CVD* cardiovascular disease, *n* number

* Values are mean ± SD or numbers (percentages)

^†^Significantly different from stable weight group (*p* for the difference <0.05)

^‡^Significantly different from progressive weight loss group (*p* for the difference <0.05)

^§^Significantly different from progressive weight gain group (*p* for the difference <0.05)

## Discussion

In our prospective population-based cohort study of middle-aged and older adults followed every 4 years for over 22 years, we examined the development of different BMI trajectories prior to the diagnosis of CVD. By using latent class trajectory analysis, we found three distinct groups of BMI change among individuals who were diagnosed with CVD during follow-up. The majority of individuals (87.8 %) who developed CVD had stable BMI levels over time. These individuals were classified into the “stable weight” group. A small group of individuals who experienced CVD during follow-up (6.4 %), which we refer to as the “progressive weight gain” group, showed a progressive increase in their mean BMI level. The third group of individuals with a CVD event (5.8 %), named the “progressive weight loss” group, experienced a decrease in their mean BMI level during follow-up. Our analysis revealed different patterns of change in other cardio-metabolic risk factors including waist circumference, HDL cholesterol, and glucose between the identified BMI trajectories. This finding further highlights that CVD is a heterogeneous disease with different pathophysiological pathways.

In general, the use of BMI as an accurate anthropometric measure in association with CVD and mortality among the elderly population has been challenged [19–21]. Recent data among middle-aged and elderly populations has demonstrated that the magnitude of relationship between elevated BMI levels and CVD weakens with age [22]. However, most of studies classified BMI into pre-defined categories which are currently debatable in relation to mortality [23]. Such an approach may also cause misclassification of individuals, especially those close to the cut-points for classification [24]. Instead of studying changes in pre-defined BMI categories, we chose to define subgroups of BMI change over time using latent class trajectory analysis. This type of statistical method is useful to explore heterogeneous growth patterns that would not be identified using conventional methods. Indeed, latent class trajectory analysis is more flexible, because it models group-specific average patterns of change in BMI during follow-up. Our latent class trajectory analysis indicated that among the majority of individuals who developed CVD during the follow-up, the mean BMI levels remained fairly stable (the “stable weight” group). Overall, within the “stable weight” group, the mean values for other cardio-metabolic risk factors also remained fairly stable overtime and their levels were mostly within the normal clinical range. In this group, we only observed a slight increase in the mean waist circumference and a decrease in mean HDL cholesterol levels before the CVD diagnosis. However, the predicted 10-year CVD risk, which combines several cardio-metabolic risk factors into a single risk score [15], showed an increase among the “stable weight” group, indicating that this subgroup was at high risk for developing CVD. This finding highlights that BMI is not a good predictor of CVD risk among middle-aged and elderly individuals [19, 22] and that a combination of multiple cardio-metabolic risk factors should be considered [15, 18].

The second group of individuals who developed CVD during follow-up, the “progressive weight gain” group, had a mean BMI level in the range of class II obesity (35–40 kg/m^2^) at the time of CVD diagnosis. During follow-up, this group showed an increase in mean waist circumference, a decrease in mean HDL cholesterol levels, and a fluctuating pattern in fasting glucose levels. Previous studies, based on a single-time measurement, have highlighted that waist circumference could play a specific role in insulin resistance and dyslipidemia [25]. The findings of our study, using multiple measurements over time, give further support to this premise by showing that the increase in waist circumference was accompanied by decrease in HDL cholesterol levels during follow-up. Furthermore, we also observed a fluctuating pattern in fasting glucose levels among the “progressive weight gain” group in our study. Whether this variability in glucose levels can be attributed to an increase in BMI levels or waist circumference needs further investigation. However, recent evidence points towards the involvement of blood glucose fluctuation in the development of vascular injury in diabetes [26]. It has been demonstrated that fluctuations in blood glucose levels can increase oxidative stress in type 2 diabetes mellitus patients [27], which results in cell dysfunction and tissue injury. The “progressive weight gain” group may therefore carry a large cardio-metabolic burden.

In young adults, weight loss is beneficial and is viewed as a positive response to lifestyle modification or medical treatment. However, among the elderly, weight loss has been associated with a high risk of mortality [17, 21, 28]. Our study comprised middle-aged and elderly individuals. Among the 3 identified BMI trajectories in our study, one distinct group showed a decline in mean BMI during follow-up (the “progressive weight loss” group). In this group, we observed a decrease in mean waist circumference as well as decreases in mean fasting glucose levels during follow-up. Despite the decreases in the mean levels of some cardio-metabolic risk factors, the predicted 10-year CVD risk showed an increase among the “progressive weight loss” group, demonstrating that this subgroup was at high risk for developing CVD. Similarly, among the group that did not develop CVD event but died of other causes (the “non-CVD mortality” group), the average BMI levels declined before death.

In our study, we were able to assess the medication data for all BMI subgroups. Interestingly, we found that the “progressive weight gain” group had the highest proportion of treatment for hypertension and hyperlipidemia. Remarkably, the trajectories of systolic blood pressure and total cholesterol in the “progressive weight gain” group were not significantly different from the “stable weight” group. Moreover, although a bit more pronounced in the “progressive weight gain” group, the predicted 10-year CVD risk increased in all 3 groups of “stable weight”, “progressive weight gain” and “progressive weight loss” during follow-up, exceeding the clinical threshold for treatment. This may suggest that the overweight and obese individuals gaining weight over time are more likely to be screened for CVD and subsequently receive medication. Notably, the “progressive weight gain” group only constituted a small proportion (around 6 %) of participants developing CVD events in our study. Therefore, treating this group has a small impact on decreasing the overall burden of CVD in total population.

Strengths of the current study include the prospective study design, large sample size, very long follow-up time, and availability of repeated measurements for BMI together with detailed data on cardio-metabolic risk factors and medication use over time. These all facilitated the analysis to create the latent classes and to estimate the trajectories of traditional cardio-metabolic risk factors. Our study overcomes the limitation of previous studies classifying BMI into pre-defined categories which is debatable among the elderly population in association with mortality [23]. Our statistical approach allows for exploring heterogeneous growth patterns that would not be identified using conventional methods. However, one disadvantage of latent class analysis is that it creates subgroups with very different sizes [29]. Therefore, comparison of subgroups, in terms of statistical power, can be difficult. Moreover, while the participants in the same group tend to be homogenous, some individual variation around the group mean is allowed.

In conclusion, latent class trajectory analysis identified three distinct patterns of BMI development prior to a CVD event. The majority of individuals who developed CVD had a stable weight during follow-up, suggesting that BMI alone is not a good indicator for identifying middle-aged and elderly individuals at high risk of CVD. Moreover, the accompanying trajectories of waist circumference, HDL cholesterol, and glucose differed between the identified BMI subgroups, further highlighting that CVD is a heterogeneous disease with different pathophysiological pathways.

## Electronic supplementary material

Below is the link to the electronic supplementary material.

Supplementary material 1 (DOCX 755 kb)
