# Supplementary material for: Trajectories of body mass index before the diagnosis of cardiovascular disease: a latent class trajectory analysis
Source: Eur J Epidemiol. 2016 Mar 8;31:583–92. doi: 10.1007/s10654-016-0131-0 (PMC4956703; doi:10.1007/s10654-016-0131-0)
Supplement: Supplementary file 1 — Supplementary material 1 (DOCX 755 kb) [file 10654_2016_131_MOESM1_ESM.docx]

**Supplementary material**

**Patterns of obesity development before the diagnosis of cardiovascular disease**

Dhana K, van Rosmalen J, Vistisen D, Ikram MA, Hofman A, Franco OH, Kavousi M

**Table S1.** Characteristics of study participants by subgroup at their first clinical examination.

**Figure S1**. Trajectories of body mass index, waist circumference, systolic and diastolic blood pressure.

**Figure S2**. Trajectories of fasting plasma glucose, total cholesterol, HDL cholesterol, and predicted 10-year CVD risk.

**Table S1.** Characteristics of study participants at their first clinical examination.

| Characteristics* | Individuals developing CVD during follow-up (n=1748) | | | Individuals without CVD during follow-up (n=4378) | |
| --- | --- | --- | --- | --- | --- |
|  | Stable weight (n=1534) | Progressive weight gain (n=112) | Progressive weight loss (n=102) | CVD-free  (n=2184) | Non-CVD mortality  (n=2194) |
| Time before diagnosis/last visit, years | 8.1 (4.04, 13.1) | 11.2 (6.9, 14.6) | 11.9 (9.2, 15.0) | 18.7 (17.4, 19.6) | 10.6 (5.7, 22.2) |
| Women (%) | 868 (56.6) | 75 (67.0) | 66 (64.7) | 1496 (68.5) | 1282 (58.5) |
| Current smoker (%) | 400 (26.1) | 20 (18.9) | 25 (25.0) | 420 (19.4) | 574 (26.2) |
| Antihypertensive treatment (%) | 278 (18.1) | 43 (38.1) | 31 (30.8) | 286 (13.1) | 397 (18.1) |
| Anti-diabetic treatment^†^ (%) | 47 (8.0) | 9 (14.1) | 9 (12.2) | 121 (6.5) | 54 (6.0) |
| Statins treatment^†^ (%) | 79 (13.4) | 16 (23.4) | 8 (10.8) | 379 (20.1) | 93 (10.1) |
| Age, years | 71.6 ± 8.8 | 69.4 ± 8.6 | 71.9 ± 7.7 | 62.9 ± 5.4 | 72.6 ± 8.7 |
| Glucose^†^, mg/dl | 106.2 ± 22.7 | 114.6 ± 27.5 | 115.1 ± 30.7 | 104.0 ± 22.5 | 107.4 ± 27.2 |
| Cholesterol^†^, mg/dl | 225.6 ± 36.3 | 225.9 ± 35.2 | 220.3 ± 35.7 | 229.6 ± 36.9 | 221.9 ± 38.2 |
| HDL cholesterol^†^, mg/dl | 52.7 ± 14.4 | 50.2 ± 14.8 | 50.9 ± 16.8 | 56.3 ± 15.0 | 53.9 ± 17.1 |
| Systolic blood pressure, mmHg | 144.7 ± 22.3 | 146.5 ± 20.6 | 144.9 ± 21.9 | 132.6 ± 20.2 | 141.7 ± 22.3 |
| Diastolic blood pressure, mmHg | 74.6 ± 12.0 | 77.5 ± 11.0 | 73.1 ± 11.2 | 73.8 ± 10.6 | 73.7 ± 12.0 |
| Body mass index, kg/m^2^ | 25.9 ± 3.2 | 31.7 ± 4.8 | 30.1 ± 4.2 | 26.2 ± 3.6 | 26.2 ± 3.9 |
| Waist circumference, cm | 90.1 ± 10.4 | 99.9 ± 13.2 | 97.2 ± 10.3 | 88.1 ± 10.9 | 91.1 ± 11.2 |

*Values are mean ± SD, numbers (percentages), or median (IQR) .

Abbreviations: HDL, high density lipoprotein; CVD, cardiovascular disease; n, number.

† Fasting measurements of Lipids and glucose and treatment were available in the third, fourth and fifth visits of the original Rotterdam Study cohort (N=3529).

The mean values of the characteristics of study participants in Table S1 are based on single measures at the baseline/first visit in the Rotterdam Study. Therefore, some differences from the predicted mean values, i.e. the results of the latent class trajectory analysis presented in the Figures 2 and 3, might be observed.

**Figure S1:** Trajectories of body mass index, waist circumference, systolic and diastolic blood pressure

**
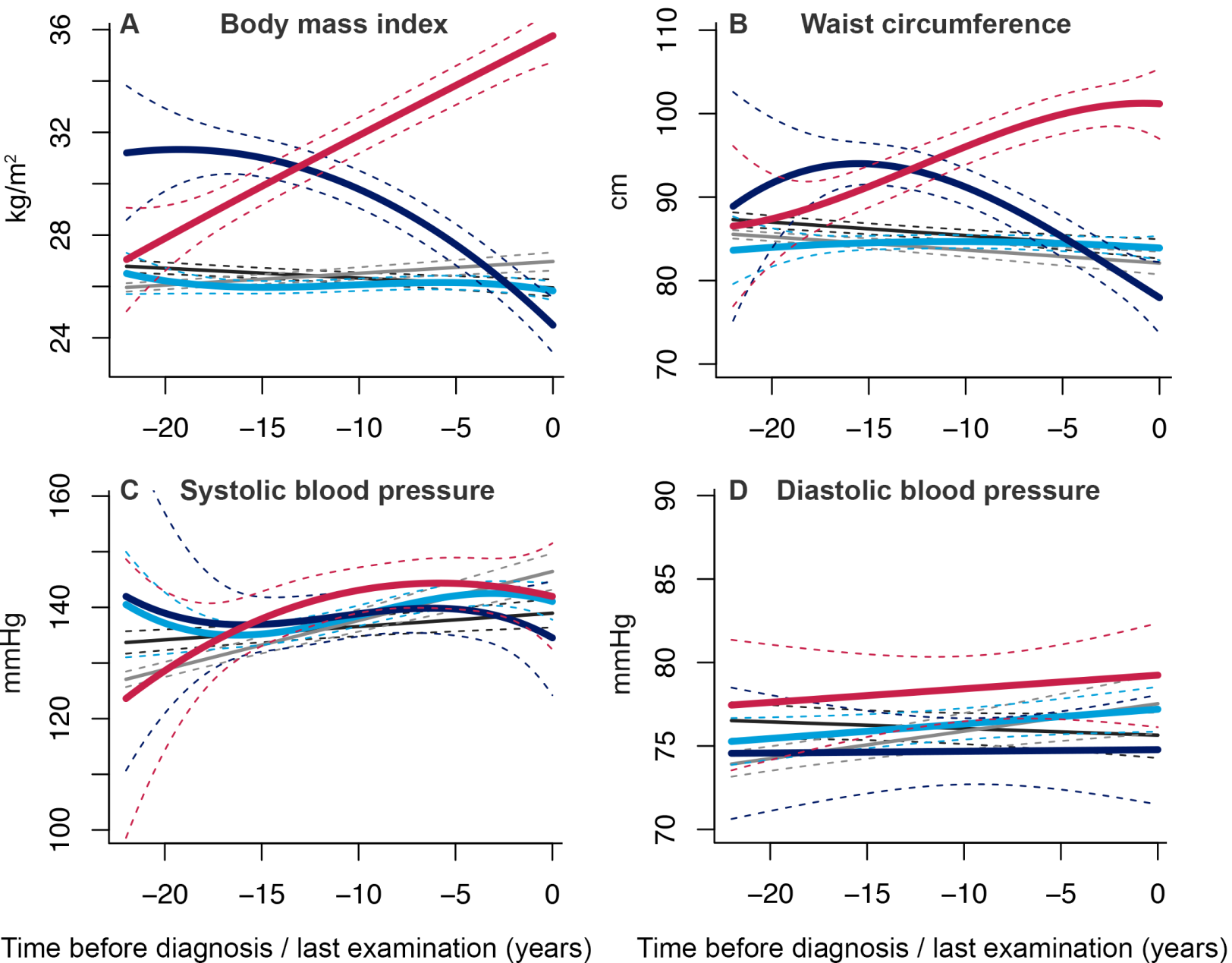
**

Trajectories for risk factors during 22 years of follow-up until diagnosis of CVD, death or censoring from the study. The figures represent a hypothetical woman of 65 years old. Trajectories for blood pressure represent a person on anti-hypertensive treatment.

Light blue: “stable weight” (including 87.8% of CVD patients); dark blue: “progressive weight loss” (including 5.8% of CVD patients); red: “progressive weight gain” (including 6.4% of CVD patients); gray: “CVD-free”; black: “non-CVD mortality” groups.

**Figure S2:** Trajectories of fasting plasma glucose, total and HDL cholesterol, and ACC/AHA 10 year CVD risk

**
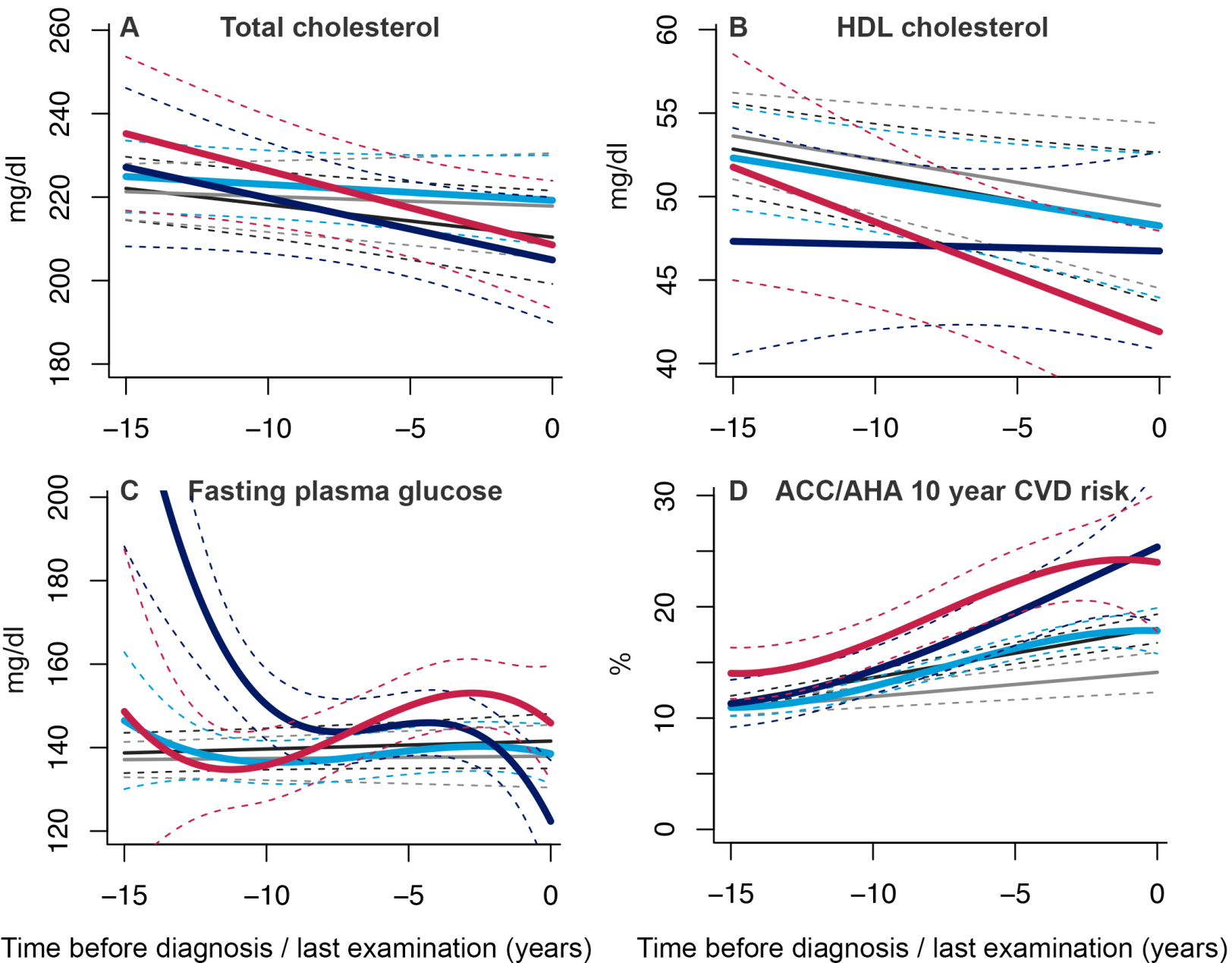
**

Abbreviation: HDL cholesterol, high-density lipoprotein cholesterol; ACC/AHA, American College of Cardiology/American Heart Association

Trajectories for risk factors during 15 years of follow-up until diagnosis of CVD, death or censoring from the study. The figures represent a hypothetical woman of 65 years old, on lipid- or glucose-lowering treatment during 15 years of follow-up until diagnosis of CVD, death or censoring from the study.

Light blue: “stable weight” (including 87.8% of CVD patients); dark blue: “progressive weight loss” (including 5.8% of CVD patients); red: “progressive weight gain” (including 6.4% of CVD patients); gray: “CVD-free”; black: “non-CVD mortality” groups.
